# In vitro effects of levosimendan on muscle of malignant hyperthermia susceptible and non-susceptible swine

**DOI:** 10.1186/s12871-018-0644-z

**Published:** 2018-12-03

**Authors:** Frank Schuster, Stephan Johannsen, Susanne Isbary, Ismail Türkmeneli, Norbert Roewer

**Affiliations:** Department of Anesthesia and Critical Care, University of Wuerzburg, Oberduerrbacher Str. 6, D-97080 Wuerzburg, Germany

**Keywords:** Malignant hyperthermia, Levosimendan, Muscle, Pigs, In vitro contracture test

## Abstract

**Background:**

The calcium sensitizer levosimendan is increasingly used to improve hemodynamics in patients with acutely decompensated heart failure. By binding to cardiac troponin C the conformation of the calcium-troponin C complex is stabilized, which leads to acceleration of actin-myosin crossbrigde formation and increased force generating capacity of muscle fibers. Besides indications in cardiac failure, beneficial effects of levosimendan in skeletal muscle disorders are currently evaluated. The aim of this study was to investigate differential effects of levosimendan on skeletal muscle of pigs with and without susceptibility to malignant hyperthermia (MH) in order to identify possible risks of this emerging drug for patients with predisposition to MH.

**Methods:**

Muscle bundles of 17 pigs (9 MH susceptible (MHS); 8 MH non-susceptible (MHN)) were excised under general anesthesia and examined in the tissue bath with increasing concentrations of levosimendan (0.065; 0.125; 0.5; 1.0; 10 and 50 μg/ml). Baseline tension and twitch force were monitored continuously. Data are presented as median and interquartile range. Statistical evaluation was performed using D’Agostino & Pearson test for normal distribution and student’s t test and 2-way ANOVA for differences between the groups. *P* < 0.05 was considered significant.

**Results:**

There were no differences between the groups concerning length, weight, initial twitch force and pre-drug resting tension of the investigated muscle strips. After an initial decrease in both groups, twitch amplitude was significantly higher in MHN (− 3.0 [− 5.2–0.2] mN) compared to MHS (− 7.5 [− 10.8- -4.5] mN) (*p* = 0.0034) muscle at an applied levosimendan concentration of 50 μg/ml. A marked increase in resting tension was detected following levosimendan incubation with 50 μg/ml in MHS muscle bundles (3.3 [0.9–6.1] mN) compared to MHN (− 0.7 [− 1.3–0.0] mN) (*p* < 0.0001).

**Conclusions:**

This in vitro investigation revealed the development of significant contractures in muscle bundles of MHS pigs after incubation with levosimendan. However, the effect appeared only at supra-therapeutic concentrations and further research is needed to determine the impact of levosimendan on MHS individuals in vivo.

## Background

Levosimendan, a calcium sensitizer and adenosine triphosphate dependent potassium channel opener mainly used for treatment of acute heart failure, improves myocardial contractility and enhances the sensitivity of myofilaments to calcium. The increase of cardiac inotropy is mediated through the binding to slow skeletal and cardiac troponin C [1, 2], while associated vasodilatation and cardioprotective effects are based on the interaction of potassium depended adenosine triphosphate channels with cardiac mitochondria and vascular smooth muscle [3]. Besides the positive inotropic modulation of cardiac function, there is growing evidence for beneficial effects of levosimendan on further organ systems. For instance, improvement of contractility of slow-twitch muscle fibers and neuromechanical efficiency of the diaphragm in animals and humans was observed following levosimendan application [4, 5]. Furthermore, a positive inotropic effect in skeletal muscle diseases was detected in vitro for calcium sensitizers due to an increased calcium mediated muscular contraction [6]. On the contrary, a pre-existing imbalance of calcium homeostasis might be associated with severe side effects for the patient, if calcium-modulating or sensitizing drugs are used.

In animals and humans susceptible to malignant hyperthermia (MH) exposure to triggering agents like volatile anesthetics and depolarizing muscle relaxants may lead to a life-threatening uncontrolled sarcoplasmic calcium release, resulting in hypoxemia, hypercapnia, tachycardia, muscular rigidity, lactic acidosis, hyperkalemia and hyperthermia. Mutations in the gene of the ryanodine receptor subtype 1 (RYR1) or of the dihydrophyridine receptor (CACNA1S) have been identified as genetic cause for the pathologic alteration of intracellular calcium handling [7]. Although the drug-induced deterioration of calcium homeostasis by calcium efflux from the sarcoplasmic reticulum represents the main pathomechanism of a developing MH crisis, different molecular pathways leading to an increase in intracellular calcium concentration may influence the metabolic state in MH-susceptible (MHS) muscle cells. Hence, administration of calcium modulating or sensitizing drugs might result in an unpredictable muscular response in individuals susceptible to MH.

In the present study, the authors investigated differential effects of the calcium sensitizer levosimendan on skeletal muscle bundles of MHS and MH-non-susceptible (MHN) pigs, hypothesizing that with clinically used levosimendan concentrations no significant muscular contractures would occur in MHS or MHN muscle bundles in vitro, excluding a possible risk of this emerging drug for MHS individuals.

## Methods

### Experimental protocol

With approval of the local animal care committee (Government of Unterfranken, Wuerzburg, Germany, No. 39/13), homozygous MHS and MHN Pietrain pigs were investigated. The pigs were purchased from a local farmer (Farm Lippert, Euerdorf, Germany) and were derived from several long-standing colonies. All the pigs used were related to one another to varying degrees up to first degree. MH susceptibility or wild type was determined by DNA analysis (GeneControl GmbH, Poing, Germany) prior to the investigation. Homozygous cysteine for arginine substitution at position 615 of RYR1 indicating MH susceptibility was proven in nine pigs while eight animals were homozygous negative for this mutation.

After insertion of an intravenous line into an ear vein, general anesthesia was induced by application of fentanyl (0.01 mg/kg) (Janssen, Neuss, Germany) and thiopental (10 mg/kg) (Nycomed, Konstanz, Germany). Afterwards, the trachea was intubated without administration of a muscle relaxant using an orotracheal tube 9.0 mm internal diameter (Teleflex Medical GmbH, Kernen, Germany). The pigs were mechanically ventilated with an inspiratory oxygen fraction of 0.3. Ventilator settings were adjusted to keep an end-tidal PCO_2_ of 32–39 mmHg (respiratory-rate: 10–16 breaths/min; tidal volume: 8–12 ml/kg; positive end-expiratory pressure: 5 mmHg). Anesthesia was maintained by continuous intravenous administration of thiopental (10 mg/kg/h) and fentanyl (0.01–0.02 mg/kg/h). Radiant heat application and warming blankets were used to ensure normothermia. Vital parameters were monitored continuously by invasive blood pressure monitoring in the tibial artery and by peripheral oxygen saturation, electrocardiography, and rectal temperature measurements.

After induction of general anesthesia, muscle specimens were excised from the left distal biceps femoris muscle, placed in carboxygenated (95% oxygen, 5% carbon dioxide) Krebs-Ringer’s solution (NaCl 118.1 mM; KCl 3.4 mM; CaCl_2_ 2.5 mM; MgSO_4_ 0.8 mM; KH_2_PO_4_ 1.2 mM; NaHCO_3_ 25.0 mM; Glucose 11.1 mM) (Pharmacy of the University Hospital, Wuerzburg, Germany) and immediately transported to the laboratory. Following preparation of the excised muscle into single muscle strips, length and wet weight of each muscle bundle was measured. For in vitro contracture test (IVCT) investigations, single muscle strips were mounted vertically in the experimental bath perfused with carboxygenated Krebs-Ringer’s solution at 37 °C, fixed to an isometric force transducer (Hugo Sachs, Type 809, March, Germany) and stimulated electrically with a supra-maximal square wave stimulus at 1 ms duration and a frequency of 0.2 Hz (Hugo-Sachs-Elektronik, Type 215/I, March, Germany). Resting tension and twitch of the muscle strips were recorded continuously by a digital recording system (HSE-ACAD Software, Hugo Sachs, March, Germany). Only muscle specimens with initial twitch response of at least 10 mN were included in the investigation. Two different muscle bundles per animal were analyzed. After stable conditions were reached, muscle bundles were incubated successively with increasing concentrations (0.065, 0.125, 0.5, 1.0, 10 and 50 μg/ml) of a commercially available levosimendan preparation (Orion Pharma GmbH, Hamburg, Germany) at 3 min intervals. To ensure sufficient viability of the muscle strips, investigations were performed within 3 h after muscle harvest.

After completion of the experiments, the pigs were euthanized in deep anesthesia by thiopental bolus application (0.1 g/kg).

#### Statistics

Data are presented as median and interquartile range. A quasi-randomization was given by the availability of the animals independently from the MH diagnosis. Statistical evaluation was performed using D’Agostino & Pearson test for normal distribution and student’s t-test (length and weight of muscle strips, pre-drug resting tension and twitch force, maximum contracture) or 2-way-ANOVA with post-hoc Sidak’s test for multiple comparisons (changes of twitch force, developing contractures) for differences between MHS and MHN groups. *P* < 0.05 was considered statistically significant.

## Results

The investigated muscle specimens did not differ between the groups regarding length (MHS: 22 [20–24] vs. MHN: 24 [21–24] mm; *p* = 0.40), weight (MHS: 920 [820–1040] vs. MHN: 920 [740–1070] mg; *p* = 0.26), initial twitch force (MHS: 25.1 [12.9–32.3] vs. MHN: 27.1 [16.5–31.7] mN; *p* = 0.84) and pre-drug resting tension (MHS: 10.4 [8.5–17.3] vs. MHN: 12.6 [7.7–20.6] mN; *p* = 0.64). One of the two investigated muscle bundles per animal had to be excluded from the study because they failed to reach the pre-assigned minimum twitch force of 10 mN in three MHS pigs and one MHN pig.

**Fig. 1:**
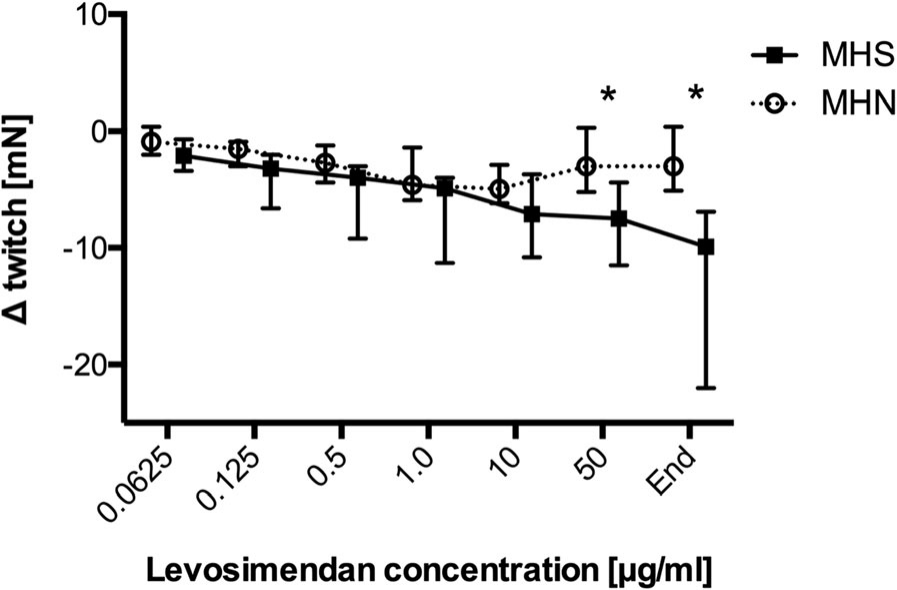
Maximum twitch force after application of increasing levosimendan concentrations in MHS and MHN muscle.Data are presented as median and quartiles; *n* = 7 for MHS; *n* = 12 for MHN; *P < 0.05 for differences between MHS and MHN. MHS: malignant hyperthermia susceptible; MHN: malignant hyperthermia non-susceptible

**Fig. 2:**
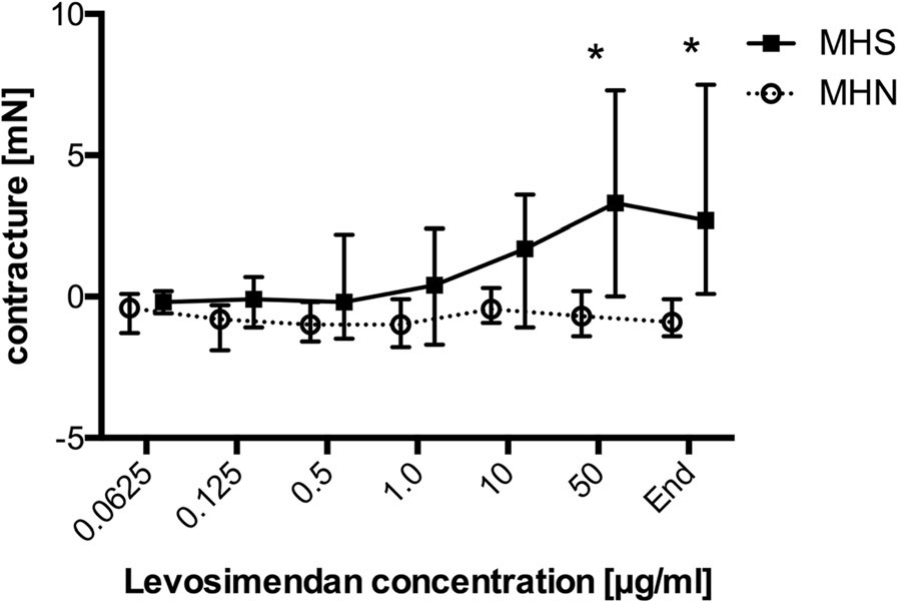
Muscular contracture in comparison with baseline resting tension after levosimendan application in MHS and MHN. Data are presented as median and quartiles; *n* = 7 for MHS; n = 12 for MHN; *P < 0.05 for differences between MHS and MHN. MHS: malignant hyperthermia susceptible; MHN: malignant hyperthermia non-susceptible

Incrementally increasing doses of levosimendan led to significantly different muscular reactions in the two investigated groups. With cumulative levosimendan concentrations ≤10 μg/ml an initial decrease of twitch amplitude in both groups was observed (at levosimendan 0.0625 μg/ml: MHS: − 2.1 [− 3.0- -0.9] vs. MHN: − 0.9 [− 1.8–0.1] mN; *p* = 0.99; at levosimendan 10 μg/ml: MHS: − 7.1 [− 10.7- -4.2] vs. MHN: − 5.0 [− 6.0- -3.2] mN; *p* = 0.26), while at 50 μg/ml twitch amplitude was significantly higher in MHN (− 3.0 [− 5.2–0.2] mN) compared to MHS (− 7.5 [− 10.8- -4.5] mN) (*p* = 0.0034) muscle bundles (Fig. 1). In addition, no significant difference in baseline resting tension was detected between both groups at levosimendan ≤10 μg/ml (at levosimendan 0.0625 μg/ml: MHS: − 0.2 [− 0.6–0.2] vs. MHN: − 0.4 [− 1.3–0.1] mN; *p* = 0.99; at levosimendan 10 μg/ml: MHS: 1.7 [0.8–3.3] vs. MHN: − 0.5 [− 0.8–0.2] mN; *p* = 0.23). However, exposure to levosimendan 50 μg/ml induced a significant higher muscular contracture in MHS compared to MHN muscle specimen (MHS: 3.3 [0.9–6.1] vs. MHN: − 0.7 [− 1.3–0.0] mN; *p* < 0.0001) (Fig. 2).

**Fig. 3:**
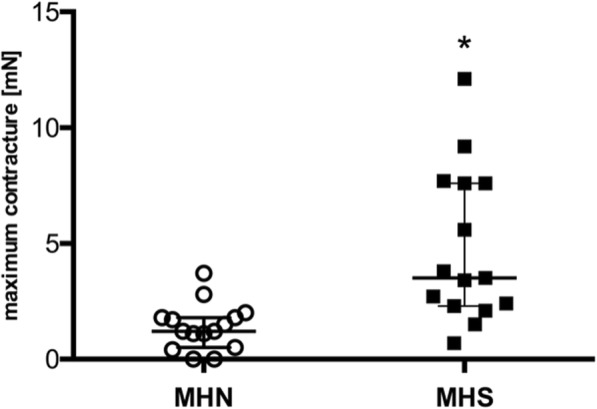
Maximum contracture in MHS and MHN muscles over the whole range of applied levosimendan concentrations. Data are presented as median and quartiles; *n* = 7 for MHS; *n* = 12 for MHN; *P < 0.05 for differences between MHS and MHN. MHS: malignant hyperthermia susceptible; MHN: malignant hyperthermia non-susceptible

Analysis of maximum developing contractures over the whole range of applied levosimendan concentrations for every individual muscle bundle, regardless at which levosimendan concentration this maximum was achieved, showed significantly stronger muscular responses in MHS animals (MHS: (3.5 [2.3–7.6] vs. MHN: 1.2 [0.5–1.8] mN; *p* = 0.0006) (Fig. 3).

## Discussion

Excessive sarcoplasmic calcium release is the underlying pathomechanism for MH crisis in susceptible individuals following exposure to trigger agents. This study investigated the influence of the calcium sensitizer levosimendan on skeletal muscle bundles of MHS and MHN swine in-vitro. Our results indicated that incubation with supra-therapeutic levosimendan doses leads to significant muscular contractures and decrease of twitch amplitude in MHS but increased twitch force in MHN muscle.

Based on MH associated mutations in the RYR1 or CACNA1S gene, triggering agents induce an excessive calcium efflux from the sarcoplasmic reticulum, resulting in a hypermetabolic state of the skeletal muscle cell, increased mitochondrial energy turnover and eventually metabolic failure. To date, various different MH causative mutations have been identified in humans, while in pigs MH phenotype is related to the homozygous mutation Arg615Cys in the RYR1 gene [8]. Thus, the porcine model is an established experimental set-up to investigate the pathophysiology of MH.

IVCT is the gold standard for diagnosing MH susceptibility. In vitro incubation with calcium releasing drugs like volatile anesthetics or caffeine results in a pathological muscular contracture above a defined diagnostic threshold in individuals susceptible to MH. Moreover, IVCT serves as a solid model to analyze pathophysiological changes during an MH reaction in-vitro [9].

While MH triggering agents increase intracellular calcium concentrations by interaction with sarcoplasmic calcium release channels, levosimendan binds to slow skeletal and cardiac troponin C and stabilizes the conformation of the troponin complex [10]. Slow skeletal and cardiac troponin C is also the dominant isoform in slow-twitch skeletal muscle fibers. Therefore, a levosimendan-induced enhancement of calcium sensitivity in slow-twitch skeletal muscle fibers seems reasonable. Levosimendan has been shown to induce a rise in submaximal force of slow-twitch diaphragm muscle fibers of patients with chronic obstructive pulmonary disease in-vitro and to improve diaphragm contractility of healthy volunteers in-vivo [11, 12]. However, the proportion of slow-twitch muscle fibers in skeletal muscle varies among different muscle groups and individuals. For instance, in healthy humans, the soleus muscle contains more than 80% slow-twitch fibers, while in the quadriceps muscle only 50% are slow-twitch. In contrast, the proportion of slow-twitch fibers in vigorous adult pigs varies between 12% in the vastus lateralis muscle and up to 80% in postural muscles like the vastus intermedius muscle. In the biceps femoris muscle fiber distribution is rather homogeneous with a proportion of slow-twitch fibers of approximately 60% [13]. Therefore, biceps femoris muscle was selected for this investigation, assuming that this mainly slow-twitch muscle is particularly suitable for analyzing a possibly impact of levosimendan on the muscular response in MHS and MHN pigs.

Assessment of pharmacokinetics of intravenous levosimendan after a single bolus application of 2 mg levosimendan revealed a plasma concentration of 0.3 μg/ml in healthy male volunteers [14]. To achieve sufficient tissue levels to provoke a reaction, in our study muscle bundles of MHS and MHN pigs were incubated with incremental concentrations of levosimendan starting from 0.0625 μg/ml up to 50 μg/ml. A 3 min interval of exposure to each investigated levosimendan concentration was predefined, similar to the protocol for IVCT issued by the European MH Group [9].

To our surprise, muscle bundles of MHS pigs developed significantly stronger contractures at levosimendan levels of 50 μg/ml while twitch amplitude decreased significantly compared to MHN. This pathological contracture might be explained by a moderate increase of intracellular calcium levels caused by phosphodiesterase (PDE) inhibition as previously observed after exposure to supra-therapeutic levosimendan concentrations. The effects of levosimendan as selective PDE-III inhibitor outweighed the calcium sensitizing effects at higher concentrations [15]. However, these effects seem irrelevant within the limits of recommended therapeutic plasma levels [16]. Based on these findings our observations are in line with previous studies indicating that pharmacological inhibition of PDE-III by enoximone leads to contracture development in muscle bundles of MHS but not of MHN pigs in-vitro [17].

Maximum individual contractures were significantly higher in MHS compared to MHN muscle. Both, contracture development and decrease of twitch force may reflect a progressive intracellular metabolic breakdown in MHS muscles, which might be further amplified by preexisting higher resting calcium levels in the skeletal muscle of MHS pigs [18]. In contrast, MHN muscles showed a significant rise of the twitch amplitude suggesting an increase in muscular force caused by levosimendan. However, the observed effects were only detected at concentrations, which exceeded the therapeutic plasma levels by at least 150 times.

Interestingly, in MHS pigs conformational changes of cardiac troponin C and its integration into cardiac myofibrils were reported after levosimendan, which might affect the calcium dependency of myocardial contractility in the course of an MH episode [19]. Whether similar reactions in skeletal muscle of MHS individuals affect intracellular calcium homeostasis and lead to muscular contractions remains unclear so far.

## Conclusions

In conclusion, the results of our investigation demonstrate that levosimendan dose-dependently causes significant contractures in MHS but not in MHN muscle in-vitro. Necessary levosimendan concentrations to induce this effect highly exceeded therapeutic plasma levels. Based on these observations, it seems unlikely that levosimendan at clinically relevant doses provokes MH crisis and poses a risk to susceptible individuals.

## Abbreviations

ANOVA: Analysis of variance
CACNA1S: Dihydrophyridine receptor
IVCT: In vitro contracture test
MH: Malignant hyperthermia
MHN: Malignant hyperthermia-non-susceptible
MHS: Malignant hyperthermia-susceptible
RYR1: Ryanodine receptor subtype 1
